# Supplementary material for: A Partially Phase-Separated Genome Sequence Assembly of the Vitis Rootstock ‘Börner’ (Vitis riparia × Vitis cinerea) and Its Exploitation for Marker Development and Targeted Mapping
Source: Front Plant Sci. 2020 Mar 4;11:156. doi: 10.3389/fpls.2020.00156 (PMC7064618; doi:10.3389/fpls.2020.00156)
Supplement: Supplementary file 1 [file DataSheet_1.docx]

**Supplementary Figure 1**. BoeWGS1.0 contigs classified according to their avearge alignment depth.
